# PanDepth, an ultrafast and efficient genomic tool for coverage calculation

**DOI:** 10.1093/bib/bbae197

**Published:** 2024-05-02

**Authors:** Huiyang Yu, Chunmei Shi, Weiming He, Feng Li, Bo Ouyang

**Affiliations:** National Key Laboratory for Germplasm Innovation and Utilization of Horticultural Crops, College of Horticulture and Forestry Sciences, Huazhong Agricultural University, No. 1 Shizishan Street, Hongshan District, Wuhan 430070, Hubei Province, China; Key Laboratory for Vegetable Biology of Hunan Province, Engineering Research Center of Education, Ministry for Germplasm Innovation and Breeding New Varieties of Horticultural Crops, College of Horticulture, Hunan Agricultural University, No. 1 Nongda Road, Furong District, Changsha, 410128, Hunan Province, China; National Key Laboratory for Germplasm Innovation and Utilization of Horticultural Crops, College of Horticulture and Forestry Sciences, Huazhong Agricultural University, No. 1 Shizishan Street, Hongshan District, Wuhan 430070, Hubei Province, China; BGI Research, Yumin street, Yazhou District, Sanya, 572025, Hainan Province, China; National Key Laboratory for Germplasm Innovation and Utilization of Horticultural Crops, College of Horticulture and Forestry Sciences, Huazhong Agricultural University, No. 1 Shizishan Street, Hongshan District, Wuhan 430070, Hubei Province, China; National Key Laboratory for Germplasm Innovation and Utilization of Horticultural Crops, College of Horticulture and Forestry Sciences, Huazhong Agricultural University, No. 1 Shizishan Street, Hongshan District, Wuhan 430070, Hubei Province, China

**Keywords:** ultrafast, coverage quantification, sequencing data

## Abstract

Coverage quantification is required in many sequencing datasets within the field of genomics research. However, most existing tools fail to provide comprehensive statistical results and exhibit limited performance gains from multithreading. Here, we present PanDepth, an ultra-fast and efficient tool for calculating coverage and depth from sequencing alignments. PanDepth outperforms other tools in computation time and memory efficiency for both BAM and CRAM-format alignment files from sequencing data, regardless of read length. It employs chromosome parallel computation and optimized data structures, resulting in ultrafast computation speeds and memory efficiency. It accepts sorted or unsorted BAM and CRAM-format alignment files as well as GTF, GFF and BED-formatted interval files or a specific window size. When provided with a reference genome sequence and the option to enable GC content calculation, PanDepth includes GC content statistics, enhancing the accuracy and reliability of copy number variation analysis. Overall, PanDepth is a powerful tool that accelerates scientific discovery in genomics research.

## INTRODUCTION

In the field of genomic research, the accurate and efficient computation of coverage and depth is crucial for evaluating sequence data quality, detecting genetic variations, revealing copy number variations (CNVs), optimizing genome assembly, etc.

Samtools [1], BEDTools [2] and Sambamba [3] are three commonly used tools among researchers in this area. Samtools presents three distinct subcommands, namely ‘depth’, ‘coverage’ and ‘bedcov’. These subcommands respectively yield the depth of base coverage, coverage metrics for each chromosome and the count of bases within specified target regions. BEDTools employs subcommands ‘coverage’ or ‘genomecov’ to output the coverage of each region or each base. Sambamba calculates coverage using the base, region and window modes in its ‘depth’ subcommand for each base, specified region, and specific window, respectively.

Subsequently, researchers have developed specialized tools such as Mosdepth [4], Megadepth [5] and BamToCov [6], all specially designed for fast and efficient computing sequencing coverage. These tools can calculate sequencing coverage for either the entire genome or specific regions. Among them, Mosdepth has gained popularity due to its more than 2-fold improvement in efficiency when calculating 30× human sequencing coverage.

However, with the rapid technological advancements and decreasing cost of sequencing, there has been a significant increase in sequencing samples and depth. Consequently, these existing tools are confronted with challenges related to time and memory consumption, rendering them incapable of efficiently performing coverage statistics on large-scale sequencing data. Moreover, most of these software outputs require further processing to obtain both coverage and mean depth metrics simultaneously.

To overcome these limitations and provide researchers with a faster and more efficient solution, we have developed PanDepth. By utilizing chromosome parallel computation and optimized data structures, PanDepth offers ultra-fast computation speed and memory efficiency for calculating coverage and mean depth from sequencing data, regardless of whether it's short reads or long reads. PanDepth accommodates sorted or unsorted BAM and CRAM-format alignment files and accepts interval files in GTF, GFF, and BED formats, or a specific window size. Furthermore, PanDepth enhances the reliability of CNV analysis through integrated GC content statistics. Overall, PanDepth empowers researchers to accelerate their genomic analysis workflow, leading to faster scientific discoveries.

## METHODS

### Overview of PanDepth workflow

**Figure 1:**
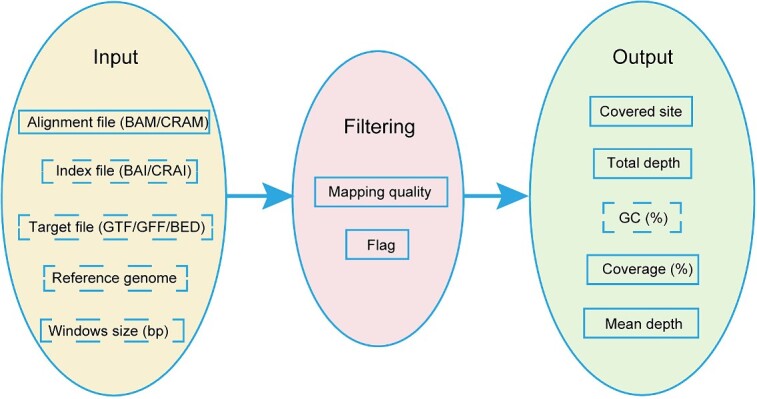
The workflow of PanDepth software. The implementation consists of three parts: input, filtering and output. The solid boxes in the ‘input’ part denote the required files, while the dotted boxes represent the optional files or options.

PanDepth is a C++ implementation, licensed under MIT and compatible with Linux/Unix and Mac operating systems. It utilizes HTSlib [7] for alignment file parsing and accepts binary compressed alignment files in BAM or CRAM format via the ‘-i’ parameter. Reads with alignment quality below a specified threshold can be excluded using the ‘-q’ parameter, and reads with specific flags can be excluded using the ‘-f’ parameter. By default, reads with flags indicating unmapped, secondary alignment, quality control failures and optical duplicates are filtered. Sequencing coverage statistics for specific regions can be obtained by providing GFF/GTF or BED files through the ‘-g’ or ‘-b’ parameters, or a specific window size through the ‘-w’ parameters, respectively. The result is reported in an output file with metrics including Covered site, Total depth, Coverage (%) and Mean depth for each chromosome, specific gene, region or window size. Additional inclusion of GC content analysis in the output can be enabled by specifying the reference genome sequence with the ‘-r’ parameter and using the ‘-c’ parameter. When the ‘-t’ parameter specifies two or more threads and index files are present, PanDepth employs parallel computing to perform sequencing coverage calculations. The program distributes the workload across multiple threads, resulting in improved efficiency (Figure 1).

### Calculation of coverage and mean depth

PanDepth can process each chromosome independently. When processing each chromosome, the method of sliding 1 MB windows is used to process each region on the chromosome. In this way, only a 1 MB integer array is needed when applying for memory, thus greatly reducing the calculation memory. The specific calculation of base coverage depth is mainly by analyzing the alignment position and cigar label of each read. In addition to calculating coverage and mean depth for each chromosome in the genome, PanDepth can also extract reads from target regions using an index file and provide coverage and mean depth specifically for those regions. When using a GFF or GTF file as input for target regions, PanDepth merges the coding sequencing or exon regions that require calculation and reports coverage information for each gene, facilitating presence/absence variation and CNV analysis.

### Accelerated method of PanDepth

PanDepth distinguishes itself from Mosdepth, Megadepth and BamToCov with its approach to acceleration. Instead of relying solely on multithreading with HTSlib [7], which has limited efficiency improvements beyond four threads, PanDepth employs a chromosome-based parallel computing method to enhance performance. By reading the header information of the alignment files, PanDepth sorts the chromosomes based on their lengths and divides them into equally sized groups by the user-specified number of threads. When the number of threads specified by the user matches the number of chromosomes, the coverage calculation is performed simultaneously for all chromosomes. If the specified number of threads exceeds the number of chromosomes, PanDepth assigns the remaining threads to the chromosomes in descending order of length, allowing multithreaded reading and decompression with HTSlib [7].

To expedite the reading speed of CRAM format alignment files, PanDepth decodes only essential information such as FLAG, RNAME, POS, MAPQ and CIGAR from the CRAM files.

### Evaluation of computational time and memory

To evaluate the performance of PanDepth, we assessed the calculation time and memory requirements of PanDepth using Illumina (short reads) and PacBio CLR (long reads) sequencing data from a previous study on pepper [8]. We extracted 150 GB of sequencing data from both short reads and long reads, and subsequently aligned them to the pepper genome (which consists of 12 chromosomes and has a size of approximately 3 GB) using BWA (v0.7.17) [9] and minimap2 (v2.26) [10]. The resulting alignment files were converted into BAM and CRAM formats using Samtools [1].

We compared PanDepth (v2.19) with established software tools, including Samtools (v1.16.1), BEDTools (v2.31.0), Sambamba (v1.0.0), Mosdepth (v0.3.5), Megadepth (v1.2.0) and BamToCov (v2.7.0), evaluating their performance in terms of time and memory usage for calculating sequencing depth at different thread numbers (1, 2, 3, 4, 6, 12, 24). Samtools, BEDTools and Sambamba were utilized for calculating the coverage of the entire genome using their respective sub-commands ‘coverage’, ‘genomecov’ and ‘depth’, respectively.

Due to the limitations in multithreading for Samtools and BEDTools, these two software tools only produced single-threaded results. Additionally, Sambamba was unable to read CRAM formatted alignment files, and therefore only BAM formatted alignment files were used for coverage calculation. All evaluations were conducted on nodes with 52 cores and 192 GB of memory in 10 repeats, using the SLURM job scheduling system.

## RESULTS

### PanDepth can accelerate the statistics of coverage

We assessed the computational time and memory requirements of PanDepth, in comparison with Samtools, BEDTools, Sambamba, Mosdepth, Megadepth and BamToCov, for calculating coverage. This assessment was conducted using different read types (short reads and long reads), alignment file formats (BAM and CRAM), and thread numbers (1, 2, 3, 4, 6, 12, 24) with 150 GB of pepper sequencing data.

**Figure 2:**
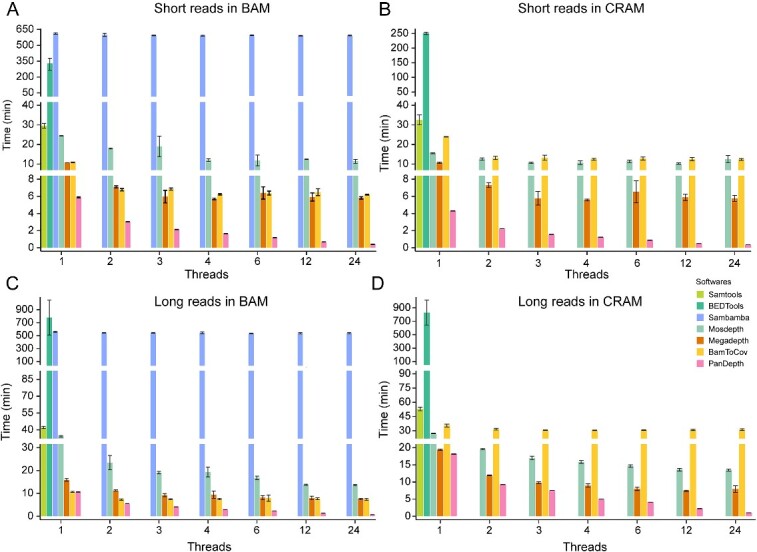
The computation time comparison of seven software tools using 150 GB sequencing reads in different numbers of threads for genome coverage calculations. (**A**) BAM file with short reads as input. (**B**) CRAM file with short reads as input. (**C**) BAM file with long reads as input. (**D**) CRAM file with long reads as input. The *X*-axis represents the number of threads and the *Y*-axis represents the computing time. The colored bars represent Samtools, BEDTools, Sambamba, MosDepth, MegaDepth, BamToCov and PanDepth, respectively. Error bars indicate standard deviation (*n* = 10).

In single-thread mode, when we perform sequencing coverage statistics in the BAM format alignment file (Figure 2A), the time required by Samtools, BEDTools, Sambamba, Mosdepth, Megadepth and BamToCov is 29.57, 321.47, 612.01, 24.4, 10.7 and 10.95 min, respectively. In contrast, PanDepth requires only 5.88 min, which is 1.82 times faster than Megadepth, 4.15 times faster than Mosdepth, 5.03 times faster than Samtools and even 54.67 and 104.1 times faster than BEDTools and Sambamba, respectively. The computation time for MosDepth and PanDepth, compared to that of BAM, decreases to 15.49 min and 4.31 min, respectively, when calculating CRAM format alignment file. PanDepth remains 3.6 times faster than MosDepth (Figure 2B). When calculating the coverage statistics of long reads (Figure 2C), the time required by Samtools, BEDTools, Sambamba, Mosdepth, Megadepth and BamToCov is 42.13, 778.44, 560.78, 34.33, 15.89, and 10.65 min, respectively. In contrast, the computation time of PanDepth is 10.62 min, which is close to BamToCov. However, it is 1.5 times faster than Megadepth, 2.19 times faster than MosDepth, 3.97 times faster than Samtools and even 52.8 and 73.3 times faster than Sambamba and BEDTools, respectively. When processing CRAM format alignment files (Figure 2D), the computation time of PanDepth is 18.16 min, which is close to the computation time of Megadepth (19.38 min), and the speed is also faster than other software.

In multi-thread mode (Figure 2), although Sambamba, Mosdepth, Megadepth and BamToCov can use multi-threading, the computation speed no longer increases when the number of threads exceeds 4. Notably, for Sambamba, multi-threading does not affect computation efficiency. However, as the number of threads specified by PanDepth increases, the computation efficiency continues to increase. Under 24 threads, the time consumed by PanDepth in calculating coverage in BAM and CRAM files of short reads is 0.41 min and 0.35 min, respectively, which are 33.05 times (12.49 min) and 30.47 times (11.4 min) faster than Mosdepth, and 14.2 times (5.82 min) and 16.68 times (5.76 min) faster than Megadepth. When calculating the BAM and CRAM alignment files of long reads under 24 threads, the computation efficiency of PanDepth remains the highest, requiring only 0.73 and 1.05 min, respectively.

### PanDepth is memory-efficient

**Figure 3:**
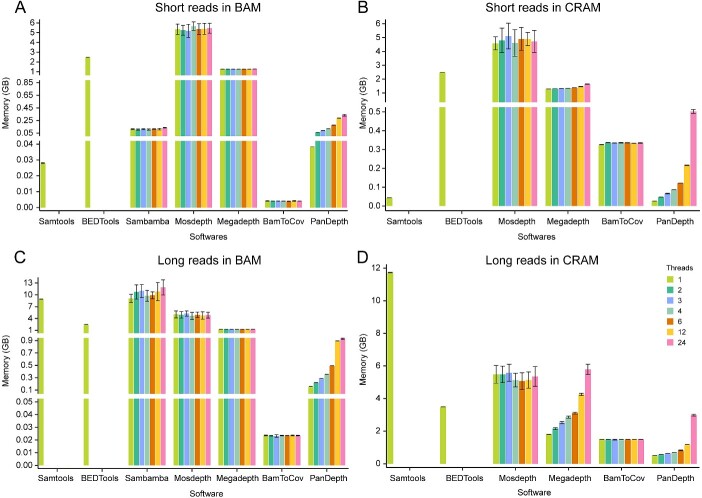
The memory consumption comparison of seven software tools using 150 GB sequencing data in different numbers of threads for genome coverage calculations. (**A**) BAM file with short reads as input. (**B**) CRAM file with short reads as input. (**C**) BAM file with long reads as input. (**D**) CRAM file with long reads as input. The *X*-axis represents the software tools, and the *Y*-axis represents memory consumption size. The colored bars represent 1, 2, 3, 4, 6, 12 and 24 threads, respectively. Error bars indicate standard deviation (*n* = 10).

Among the seven software packages we evaluated, Samtools, BamToCov and PanDepth are the most memory-efficient when conducting genomic statistics on short reads in single-thread mode, with their memory usage not exceeding 350 MB (Figure 3A). Although BamToCov consumes approximately 4 MB of memory when processing BAM files, this increases to 325 MB when analyzing CRAM files (Figure 3B). Conversely, when PanDepth processes CRAM files, its memory usage decreases from 38 to 25 MB. When performing statistics on long reads, the memory usage of Samtools for BAM and CRAM files significantly increases to 8.92 and 11.76 GB, respectively, and the memory usage of Sambamba for BAM files increases to between 9.08 GB (Figure 3C and D). In contrast, the memory usage of PanDepth for BAM and CRAM files is only 157 and 505 MB, respectively. Although the memory usage of PanDepth increases with the increase in the number of threads, it remains at a lower level compared to other software.

### The usage of PanDepth is straightforward

PanDepth can accommodate both sorted and unsorted BAM or CRAM files. For sorted BAM or CRAM files, index files are not obligatory, but when available, multi-threading can be utilized to expedite data analysis. PanDepth can also accept GTF, GFF, BED format files or specific window sizes, enabling users to compute the statistical results of particular regions. If the input consists of genomic annotation results (GTF or GFF), PanDepth will output the statistical results for each gene. To prevent the generation of excessively large output files, PanDepth does not output the base coverage for each site but instead outputs the four metrics that researchers prioritize when calculating alignment files: Covered site, Total depth, Coverage (%) and Mean depth. When the reference genome sequence is input and GC statistics (−c) are enabled, the GC content will be computed, allowing users to adjust the GC content in CNV analysis, thereby enhancing the accuracy and reliability of CNV analysis. A comprehensive comparison between PanDepth and other tools is presented in Table 1.

**Table 1:** Comparing the software tools of coverage calculation

Performance	Samtools	BEDTools	Sambamba	Mosdepth	Megadepth	BamToCov	PanDepth
*Input*							
Sorted bam	√	√	√	√	√	√	√
Sorted cram	√	√	✕	√	√	√	√
Unsorted bam	✕	✕	✕	✕	✕	✕	√
Unsorted cram	✕	✕	✕	✕	✕	✕	√
GFF	✕	✕	✕	✕	✕	√	√
GTF	✕	✕	✕	✕	✕	√	√
BED	✕	√	√	√	√	√	√
Window size	✕	√	√	√	√	✕	√
*Filtering*							
Mapping quality	√	✕	√	√	✕	√	√
Flag	√	✕	√	√	✕	√	√
*Output*							
Covered site	√	✕	✕	✕	✕	✕	√
Total depth	√	√	√	√	√	√	√
GC (%)	✕	✕	✕	✕	✕	✕	√
Coverage (%)	√	✕	✕	✕	✕	✕	√
Mean depth	√	✕	✕	√	✕	√	√

## DISCUSSION

The field of genomic research has seen a significant increase in the number of samples and sequencing depth due to rapid technological advancements and the decreasing cost of sequencing. This has posed challenges to existing tools such as Samtools [1], BEDTools [2], Sambamba [3], Mosdepth [4], Megadepth [5] and BamToCov [6] in terms of time and memory consumption, making them incapable of efficiently computing coverage statistics for large-scale, high-depth sequencing samples. Moreover, most of these tools require further processing to obtain both coverage and mean depth metrics simultaneously, adding to the computational time and complexity.

In response to these challenges, we have developed PanDepth, a tool designed to overcome the limitations of existing tools and provide researchers with a faster and more efficient solution for calculating coverage and mean depth from sequencing data, and its statistical results are completely consistent with Samtools. By utilizing chromosome parallel computation and optimized data structures, PanDepth offers ultra-fast computation speed. It accommodates sorted or unsorted BAM and CRAM-format alignment files and accepts interval files in GTF, GFF and BED formats or a specific window size, enhancing its versatility and usability in various research contexts.

Furthermore, PanDepth enhances the reliability of CNV analysis through integrated GC content statistics, providing researchers with more accurate and reliable data. This feature is particularly important as accurate CNV analysis is crucial for detecting genetic variations and revealing CNV.

In conclusion, our study introduces PanDepth as a powerful and efficient tool for calculating coverage and depth from sequencing data. Its superior computation speeds, low memory requirements and user-friendly features make it a valuable asset for genomic researchers, leading to faster scientific discoveries in their research endeavors.

**Key Points**

- PanDepth is a high-performance tool for calculating coverage in sequencing alignments, outperforming other tools in speed for both BAM and CRAM-format alignment files, regardless of read length.
- PanDepth accepts sorted or unsorted BAM and CRAM-format alignment files and GTF/GFF/BED-formatted interval files.
- PanDepth is memory efficient, making it an attractive choice for large-scale genomic data analysis.

## WEB RESOURCES

PanDepth is freely available for non-commercial research institutions. Details can be obtained from https://github.com/HuiyangYu/PanDepth. The code and results evaluating the performance of different software in this study can be accessed from https://github.com/HuiyangYu/manuscript.

## Data Availability

Any required links or identifiers for the data used in this manuscript are present in the Methods section.
